# A retrospective analysis of treatment‐related hospitalization costs of pediatric, adolescent, and young adult acute lymphoblastic leukemia

**DOI:** 10.1002/cam4.583

**Published:** 2015-12-29

**Authors:** Sapna Kaul, Ernest Kent Korgenski, Jian Ying, Christi F. Ng, Rochelle R. Smits‐Seemann, Richard E. Nelson, Seth Andrews, Elizabeth Raetz, Mark Fluchel, Richard Lemons, Anne C. Kirchhoff

**Affiliations:** ^1^ University of UtahSalt Lake CityUtah; ^2^ Intermountain HealthcareSalt Lake CityUtah; ^3^ Tufts University School of MedicineBostonMassachusetts; ^4^ Primary Children's HospitalSalt Lake CityUtah

**Keywords:** Acute lymphoblastic leukemia, hospital costs, pediatric/adolescent/young adult

## Abstract

This retrospective study examined the longitudinal hospital outcomes (costs adjusted for inflation, hospital days, and admissions) associated with the treatment of pediatric, adolescent, and young adult acute lymphoblastic leukemia (ALL). Patients between one and 26 years of age with newly diagnosed ALL, who were treated at Primary Children's Hospital (PCH) in Salt Lake City, Utah were included. Treatment and hospitalization data were retrieved from system‐wide cancer registry and enterprise data warehouse. PCH is a member of the Children's Oncology Group (COG) and patients were treated on, or according to, active COG protocols. Treatment‐related hospital costs of ALL were examined by computing the average annual growth rates (AAGR). Longitudinal regressions identified patient characteristics associated with costs. A total of 505 patients (46.9% female) were included. The majority of patients had B‐cell lineage ALL, 6.7% had T‐ALL, and the median age at diagnosis was 4 years. Per‐patient, first‐year ALL hospitalization costs at PCH rose from $24,197 in 1998 to $37,924 in 2012. The AAGRs were 6.1, 13.0, and 7.6% for total, pharmacy, and room and care costs, respectively. Average days (AAGR = 5.2%) and admissions (AAGR = 3.8%) also demonstrated an increasing trend. High‐risk patients had 47% higher costs per 6‐month period in the first 5 years from diagnosis than standard‐risk patients (*P* < 0.001). Similarly, relapsed ALL and stem cell transplantations were associated with significantly higher costs than nonrelapsed and no transplantations, respectively (*P* < 0.001). Increasing treatment‐related costs of ALL demonstrate an area for further investigation. Value‐based interventions such as identifying low‐risk fever and neutropenia patients and managing them in outpatient settings should be evaluated for reducing the hospital burden of ALL.

## Introduction

In the United States, over 3000 children under the age of 19 will be diagnosed with acute lymphoblastic leukemia (ALL) in 2015 1. ALL occurs in 35 to 40 cases per million annually 2, 3. Treatment for ALL includes intensive chemotherapy for 6 to 9 months followed by a less‐intense maintenance phase. Due to a higher risk of relapse, males receive 3 years of therapy from the start of interim maintenance versus 2 years for females 4. Therapy varies according to clinical and biological disease features and early treatment response, including assessment of minimal residual disease 4, 5, 6, 7, 8, 9. With contemporary therapy and excluding cranial irradiation, the rate of relapse after completing ALL treatment is approximately 6% 10. Chemotherapy is initiated in the hospital after diagnosis. Subsequently, patients are hospitalized for inpatient chemotherapy or due to therapy‐induced toxicities including fever and neutropenia, infection, neurotoxicity, cardiotoxicity, thrombosis, and osteonecrosis 5, 11, 12, 13, 14. Postinduction therapy has been intensified to improve survival 15. These treatment‐related and other institutional or external factors can impact treatment costs of ALL.

While there is a lack of adequate information on the costs associated with childhood ALL, pediatric and adolescent cancer‐related hospitalizations, on average, cost five times as much as hospitalizations for other pediatric conditions in the United States 16. Costs of cancer care in the United States are expected to rise faster than overall healthcare costs 17, 18, yet longitudinal cost‐related examinations of pediatric cancer are rarely conducted. Childhood cancer cost studies are often limited to charges, which is the amount billed to private insurance companies or public insurance program such as the Medicaid for the receipt of healthcare services. However, charges may not reflect the true cost of care 16, 19. Evaluations based on actual cost of care are essential for exploring value‐based models for pediatric cancer care.

This study examined treatment‐related hospital outcomes (costs adjusted for inflation, days in hospital, and admissions) among pediatric, adolescent, and young adult ALL patients treated at Intermountain Healthcare's (IH) Primary Children's Hospital (PCH), Salt Lake City, UT. Temporal changes in hospital costs, cost components (e.g., room and care, and pharmacy costs), admissions, and days were examined to understand the drivers of rising costs from 1998 to 2012. Patient and diagnostic factors (e.g., sex and treatment risk classification at diagnosis) associated with hospitalization outcomes within 5 years of diagnosis were also evaluated.

## Methods

### Patients with acute lymphoblastic leukemia

Patients were diagnosed between one and 26 years of age, from January 1, 1998–December 31, 2012. As available electronic data prior to 1998 was less comprehensive, we restricted our analysis to these years. Patients received full or part of their treatment at PCH, which serves as the sole children's hospital for Utah and six other neighboring states 20. Many of the adolescent and young adult patients with ALL are treated at PCH 21. Infants with ALL (about 3% of the sample) were excluded as they represent a distinctly high‐risk entity. Burkitt's leukemia was excluded because of a small sample size (<1% of the sample). PCH is a member of the Children's Oncology Group (COG). Therefore, patients were treated on, or according to, active COG protocols. Please see Appendix Figure S1B on ALL‐specific clinical trials implemented at PCH from 1998 to 2012. The data of patients were extracted from IH's enterprise data warehouse (EDW) and cancer registry, which reports to the Utah Cancer Registry — a National Cancer Institute's Surveillance, Epidemiology, and End Results Program site. The Institutional Review Board of the University of Utah approved this study.

### Study design

We examined hospitalization outcomes including costs, days (i.e., discharge date minus admit date), and admissions associated with in‐patient stays at PCH for our patient cohort. Hospitalization data were from January 1, 1998 to December 31, 2013 to allow for at least one year of follow‐up for our sample. An activity‐based accounting approach is used to compute costs and volume of healthcare activities delivered at PCH. Costs are calculated by taking the total operating expenses and applying them to volume metrics. Total costs are distributed across components such as (1) room and care (includes daily room and nursing costs), (2) therapy (e.g., operating room and supplies, stem cell transplant (SCT), and radiation), (3) diagnostics (lab and imaging), and (4) pharmacy (in‐patient medication). All costs were adjusted for inflation using the Producer Price Index for Hospitals with 2013 as the base year to examine real changes in costs 22. Pregnancy‐related hospitalizations (<0.5% of all hospitalizations) were excluded from our analyses 19, 23.

### Statistical analyses

Patient demographics, diagnosis, treatment, and cancer‐specific outcomes were summarized. We examined annual per‐patient hospitalization outcomes (i.e., average outcomes per hospital admission at PCH) and cost components for the first year following cancer diagnosis, typically the therapy‐intensive year, for patients diagnosed from 1998 to 2012. Per‐patient outcomes were also plotted separately for high‐ and standard‐risk ALL (refer to Appendix S1 for the definition of type of leukemia).

Longitudinal regressions examined associations between patient factors with per‐period (increments of 6 months from the date of diagnosis) costs, days, and admissions within the first 5 years of diagnosis 24, 25. These regressions modeled the variation in hospital outcomes, conditional on having nonzero outcomes, using gamma and Poisson distributions and log link for cost and days and admissions, respectively. Please see Appendix S1 for a detailed explanation of this method and for variable definitions. Independent variables in these regressions included sex, primary payer type at closest encounter to diagnosis, ALL risk (high vs. standard), radiation versus no radiation, SCT versus no SCT, relapse versus no relapse, an indicator for first period versus remaining periods, and a linear term for all periods. Adjustments were made for year of diagnosis, end‐of‐life period and timing of death in a period. Confidence intervals (95% CI) were estimated for all coefficients that depicted the ratio of dependent variable (e.g., per‐period cost) across independent variable categories (e.g., high vs. standard risk), conditional on nonzero outcomes.

The data were analyzed with the software packages Stata 13 (Stata Corporation, College Station, TX) and R 3.0.2 (The R Foundation for Statistical Computing) 26. Longitudinal regression analyses were conducted in SAS V9 (SAS Institute Inc., Cary, NC). Reported *P*‐values are two sided and considered significant at *α* = 0.05. Data are presented as medians (ranges) unless otherwise specified.

## Results

### Patient characteristics

Most patients (75.3%) were diagnosed at <10 years of age (Table 1). Females constituted 46.9% of patients and 91.0% were white. Most (71.1%) were privately insured at diagnosis and 24.8% had public insurance. The majority (63%) had standard‐risk ALL and 6.7% had T‐cell ALL. Approximately 10% of patients received at least one radiation therapy treatment, 8.7% of the patients underwent SCT, and almost all (96.2%) had surgical placement of a central venous catheter. ALL recurred in 13.3% of patients and 8.3% of died within 5 years of diagnosis. The median follow‐up time was 6.4 years (range 0.003–16 years).

**Table 1:** Characteristics of pediatric acute lymphoblastic leukemia patients

	Patients (total = 505)	
Demographics	*N*	%
Age at diagnosis (years)		
1–9	380	75.3
10–26	125	24.7
Sex		
Female	237	46.9
Male	268	53.1
Race^a^		
White	443	91.0
Other	44	9.0
Diagnosis year		
1998–2001	112	22.2
2002–2005	141	27.9
2006–2009	133	26.3
2010–2012	119	23.6
Primary payer at diagnosis^b^		
Private	359	71.1
Public	125	24.8
Uninsured	21	4.2
State of residence at diagnosis^b^		
Utah	403	79.8
Other	102	20.2
Cancer‐related		
Risk at diagnosis		
Standard	319	63.2
High	186	36.8
Leukemia lineage		
T‐cell	34	6.7
B‐cell	471	93.3
Radiation^c^		
Yes	51	10.1
No	454	89.9
Stem cell transplant^c^		
Yes	44	8.7
No	461	91.3
Central venous catheter		
Yes	486	96.2
No	19	3.8

a Due to missing data, the total number of patients for race does not sum to 505.

b Determined at the closest hospitalization encounter to the cancer diagnosis date.

c Radiation describes how many patients received at least one radiation therapy in the first 5 years of diagnosis. Stem Cell Transplant is coded in the same fashion.

### Annual average growth rates

**Figure 1:**
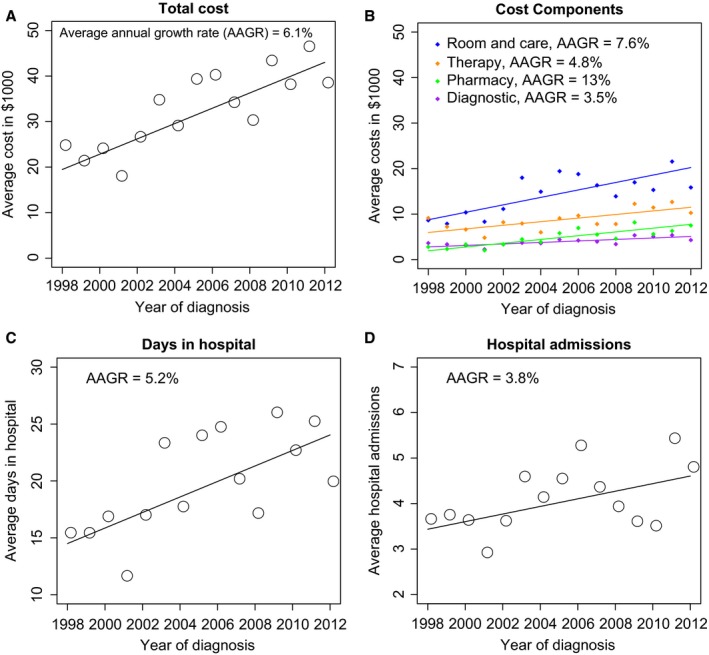
Per‐patient first‐year hospitalization outcomes for patients diagnosed from 1998 to 2012. Average hospital outcomes (i.e., cost, cost components, days and admissions) were computed by dividing the aggregate outcomes in a year by the number of patients diagnosed in that specific year. The annual growth rates for each outcome were computed as (average outcome in year (*t*)‐average outcome in year (*t* − 1))*100/average outcome in year (*t* − 1). The average annual growth rate for an outcome was computed by dividing the sum of all growth rates from 1999 to 2012 by the total number of growth rates. The lines depict fitted linear regressions for the hospital outcomes.

**Figure 2:**
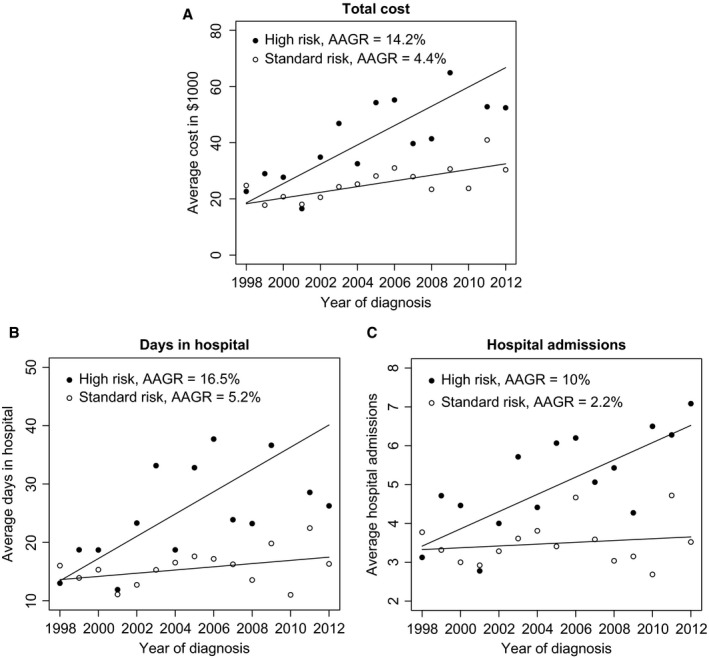
Per‐patient first‐year hospitalization outcomes for patients diagnosed from 1998 to 2012 by acute lymphoblastic leukemia risk stratification. Please refer footnote of Figure 1 for information on average per‐patient hospital outcomes and average annual growth rates computations. The lines depict linearly fitted regressions for all the hospitalization outcomes.

The average or per‐patient first‐year hospitalization costs rose from $24,197 in 1998 to $37,924 in 2012, with an annual average growth rate (AAGR) of 6.1% (Fig. 1A). The average room and care costs rose from $8646 to $15,849 (Fig. 1B; AAGR = 7.6%). Similarly, average pharmacy costs increased from $2790 to $7510 (AAGR = 13%), therapy costs rose from $9132 to $10,271 (AAGR = 4.8%), and diagnostic costs increased from $3629 to $4295 (AAGR = 3.5%). For per‐patient days in hospital (Fig. 1C, AAGR = 5.2%) and hospital admissions (Fig. 1D, AAGR = 3.8%), an upward trend was also observed. Trends in median total cost, average fixed and variable costs were similar (refer to Fig. S1C). Although not significantly different, average costs for high‐ and standard‐risk patients increased at AAGR to 14.2% (Fig. 2A) and 4.4% (Fig. 2B), respectively (*P* = 0.50). High‐risk patients also encountered more growth in hospital days (AAGR = 16.5 vs. 5.2% for standard risk, *P* = 0.71) and admissions (AAGR = 10% vs. 2.2% for standard risk, *P* = 0.47).

### Longitudinal regression results

Conditional on nonzero costs, high‐risk patients had higher per‐period costs (ratio = 1.47, 95% CI: 1.34–1.61, *P* < 0.001), spent more days in the hospital (ratio = 1.59, 95% CI: 1.39–1.83, *P* < 0.001), and had more hospital admissions (ratio = 1.54, 95% CI: 1.36–1.75, *P* < 0.001) compared to standard‐risk patients (Table 2). Patients who relapsed had over three times greater costs (ratio = 3.15, 95% CI: 2.40–4.12, *P* < 0.001) after relapse, along with hospital stays over four times longer (ratio = 4.30, 95% CI: 2.89–6.39, *P* < 0.001), and four times more admissions (ratio = 4.02, 95% CI: 2.95–5.48, *P* < 0.001) than patients who did not relapse. Patients who received SCT were likely to have greater costs (ratio = 1.84, 95% CI: 1.37–2.48, *P* < 0.001) during SCT or post‐SCT periods, and spend more days in hospital (ratio = 3.04, 95% CI: 2.18–4.24, *P* < 0.001) in comparison to patients who did not receive SCT. Compared to patients who survived, those who were nearing death were more than twice as likely to accrue higher hospitalization costs (ratio = 2.29, 95% CI: 1.65–3.19, *P* < 0.001) in the period prior to death, yet there were no significant differences in days (ratio = 1.47, 95% CI: 0.93–2.34, *P* = 0.10) or admissions (ratio = 1.05, 95% CI: 0.80–1.38, *P* = 0.72). Importantly, on average, the first 6 months costs (ratio = 3.84, 95% CI: 3.39–4.36, *P* < 0.001), days (ratio = 2.41, 95% CI = 2.01–2.89, *P* < 0.001), and admissions (ratio = 2.47, 95% CI: 2.08–2.95, *P* < 0.001) were highest than within any 6‐month period in the 5 years of diagnosis.

**Table 2:** Regression results for pediatric acute lymphoblastic leukemia patients^a^

Variables^b^	Total cost		Days in hospital		Hospital admissions	
	Ratio (95% CI)	*P*	Ratio (95% CI)	*P*	Ratio (95% CI)	*P*
Female versus Male	1.07 (0.98–1.16)	0.13	1.11 (0.98–1.25)	0.10	1.10 (0.97–1.25)	0.14
Public versus Private insurance	1.01 (0.92–1.11)	0.87	1.00 (0.87–1.17)	0.92	0.89 (0.76–1.04)	0.16
Uninsured versus Private insurance	0.85 (0.65–1.13)	0.27	1.05 (0.75–1.46)	0.79	0.90 (0.66–1.23)	0.51
High versus Standard‐risk	**1.47 (1.34–1.61)**	**<0.001**	**1.59 (1.39–1.83)**	**<0.001**	**1.54 (1.36–1.75)**	**<0.001**
Radiation	1.04 (0.85–1.28)	0.70	0.94 (0.67–1.32)	0.72	0.97 (0.76–1.22)	0.78
Stem cell transplant versus No transplant	**1.84 (1.37–2.48)**	**<0.001**	**3.04 (2.18–4.24)**	**<0.001**	1.08 (0.83–1.39)	0.58
Relapse versus No relapse	**3.15 (2.40–4.12)**	**<0.001**	**4.30 (2.89–6.39)**	**<0.001**	**4.02 (2.95–5.48)**	**<0.001**
Near death	**2.29 (1.65–3.19)**	**<0.001**	1.47 (0.93–2.34)	0.10	1.05 (0.80–1.38)	0.72
First 6 months	**3.84 (3.39–4.36)**	**<0.001**	**2.41 (2.01–2.89)**	**<0.001**	**2.47 (2.08–2.95)**	**<0.001**

a The first parts of these regressions model the probability of having nonzero hospitalizations (not shown in this table) and the second part models the variation in dependent variables (cost, days and admissions) conditional on having one or more hospitalizations. The estimated coefficients reflect the ratio of dependent variable across the covariate categories. Bolding indicates statistical significance.

b Several other variables were included in these models (not shown in the table). First period typically is the highest cost period since almost every patient is hospitalized in the first 6 months from diagnosis, so we included a dummy variable for the first period and a linear term for all the remaining periods. If a patient was lost to follow‐up in a specific period, we did partial period adjustments with respect to the duration of time the patient was followed‐up in that specific period. Diagnosis year was also included as a continuous variable in the second part of two‐part regression models. In addition, these models included an intercept and random effects for intercepts.

## Discussion

To the best of our knowledge, this is the first study to examine direct hospitalization costs for treating pediatric, adolescent, and young adult ALL. First‐year per‐patient hospitalization costs of ALL increased at an annual average growth rate of 6% from $24,197 in 1998 to $37,924 in 2012 at Primary Children's Hospital. Existing evidence indicates that survival for pediatric and adolescent ALL has increased over time 27, 28. Therefore, increasing per‐patient cost does not indicate lower effectiveness although it does demonstrate an area for further investigation. High‐risk ALL patients had 47% higher costs per period than standard‐risk patients, potentially because of the intensive therapy that high‐risk patients receive. Patients with relapsed ALL accrued almost three times greater costs per‐period than patients who did not relapse. Relapsed patients require very intensive therapy including SCTs 29, 30, and may receive newer agents/drugs 12, which are often expensive. Consistent with findings from other studies, patients nearing end‐of‐life had greater costs per‐period than those who survived 31, 32, 33. The first 6 months of ALL treatment were associated with highest costs.

Pharmacy costs grew at a faster rate than overall costs (AAGR = 13%). Drug‐acquiring costs have increased over time, which have been observed in previous studies 34, 35. Room and care costs rose at an AAGR of 8%. Certain institutional changes during our study period, such as implementation of new programs/software for managing patient data, high‐efficiency particulate arresting filtration of inpatient units and patient rooms, and patient monitoring systems might contribute to this increase. Per‐patient hospital days and admissions also increased at about 5 and 4% annually, respectively. Notably, PCH is representative of the national distribution of pediatric and adolescent cancers 36. Thus, our estimates on costs, days, and admissions may be fairly generalizable.

Our study is important because we investigate increasing costs, rather than charges, of hospitalizations associated with the treatment of ALL from the perspective of a large hospital. At PCH, much of the patient care has been shifted to outpatient clinics and home care, yet high‐risk ALL patients may be hospitalized more frequently for high‐dose methotrexate administration in an attempt to prevent relapse after remission induction therapy 37. Furthermore, treatment intensification may increase the likelihood of developing treatment‐related toxicities that may further increase the risk of hospitalizations for ALL patients. While improving health outcomes among pediatric cancer patients is the most important priority for future research, we recommend examining alternate strategies to reduce growing cancer care costs. One potential intervention is to develop better methods for identifying low‐risk fever and neutropenia 38, 39, 40, which are common reasons for hospitalizations for pediatric cancer patients, so that less costly alternatives (e.g., outpatient admissions and use of oral fluoroquinolones prophylactic antibiotics) can be evaluated 41, 42, 43.

This article has certain limitations. Our analyses were retrospective and restricted to one institution. ALL treatments implemented worldwide may define risk differently, although these risk algorithms include the NCI risk criteria used in this study. Patients in our cohort may have been hospitalized at other facilities, which could lead to an underestimation of true costs. We were unable to define treatment costs in terms of medication type (e.g., standard vs. experimental chemotherapy, supportive care medications). Likewise, we could not examine the differences in costs according to leukemia subtype (e.g., Ph+ ALL). We did not incorporate minimal residual disease in risk classification since it was not routinely used for all patients in this study 44. Due to unavailability of clinical trials electronic data, we did not evaluate the associations of costs with trials. We were unable to include other costs (e.g., clinic and outpatient lab costs, physician costs as PCH physicians are employed through the University of Utah, which has a separate accounting system, and indirect healthcare costs 45, 46, 47, 48) of treating ALL. The rural geography of the Intermountain West may affect indirect costs (e.g., cost of travel to PCH) for families in Utah. Finally, the state of Utah may not be representative of the distribution of race and ethnicity nationwide.

To conclude, we have shown that the costs of high‐risk ALL patients are 47% more, per period, than for standard‐risk patients. Value‐based interventions and outpatient supportive care should be evaluated to reduce the ALL burden. We recommend cost‐based comparisons of protocols such as Dana‐Farber Cancer Institute ALL Consortium Protocol 05‐001^49^ and 11‐001 50, St. Jude's Total Therapy Study XVI 51, and Dutch Childhood Oncology Group ALL‐11^9^, ^52^ to identify strategies for improving patient outcomes while keeping the costs low. Finally, future studies should incorporate outpatient and indirect costs to identify over‐all costs of all types of pediatric cancer.

## Conflict of Interest

Authors have no conflict of interest to report.

## Supporting information


**Appendix S1.** Methods.
**Table S1.** Acute lymphoblastic leukemia treatment protocols followed by the primary children's hospital from 1998 to 2012.^a^
**Figure S1.** Per‐patient first‐year hospitalization cost for pediatric, adolescent, and young adult acute lymphoblastic leukemia from 1998 to 2012.
