# Supplementary material for: A retrospective analysis of treatment‐related hospitalization costs of pediatric, adolescent, and young adult acute lymphoblastic leukemia
Source: Cancer Med. 2015 Dec 29;5(2):221–9. doi: 10.1002/cam4.583 (PMC4735779; doi:10.1002/cam4.583)
Supplement: Supplementary file 1 — Appendix S1. Methods. Table S1. Acute lymphoblastic leukemia treatment protocols followed by the primary children's hospital from 1998 to 2012.a Figure S1. Per‐patient first‐year hospitalization cost for pediatric, adolescent, and young adult acute lymphoblastic leukemia from 1998 to 2012. [file CAM4-5-221-s001.docx]

**Supplemental Appendix**

**Appendix S1: Methods**

**Statistical Analysis**

Summary statistics of patient characteristics were estimated. Demographics at diagnosis included age, sex, race, year of diagnosis, primary payer at the closest encounter to date of diagnosis (public, private or uninsured), and state of residence (Utah or other). Diagnosis information included white blood cell (WBC) count at diagnosis and acute ALL immunophenotype (B-cell or T-cell ALL lineage). Treatment information included whether patients received radiation therapy, a stem cell transplant (SCT), or central venous catheter. Cancer-specific outcomes included ALL relapse and mortality.

Using the National Cancer Institute (NCI) criteria,^1,2^ children ages 1 to younger than 10 years old at diagnosis with a WBC count<50,000 /uL were designated standard-risk and patients 10 years or older, or of any age with a WBC count≥50,000 /uL at diagnosis, were designated high-risk. Children with T-lineage leukemia were included in the high-risk category. Data on demographics, cancer diagnosis, treatment, cancer-specific health outcomes, and hospitalizations at PCH were extracted from IH’s EDW and cancer registry. EDW also reported the last known date for patients seen in IH facilities.

**Regression Method**

We conducted longitudinal regression analyses to examine associations between patient characteristics and other factors with hospitalization costs, days and admissions within the first five years of diagnosis. We used the Olsen and Schafer’s two-part longitudinal random-effects model to account for semi-continuity, censoring and differential utilization in hospitalization outcomes.^3,4^ Data for the regressions were constructed with one observation for each patient-period, where periods were defined as six-month increments over the five years following cancer diagnosis. Each patient had at most 10 periods (0-6, up to 55-60 months after diagnosis), or fewer periods if they were censored. We used the admission date to sum hospitalization outcomes (costs, days and admissions) at the patient-period level. If patients had no hospitalizations in a period, hospitalization was indicated as a “0” for that period.

For each hospitalization outcome (i.e., cost, days and admissions), we first modeled the probability of having at least one hospital encounter at PCH for each patient-period using a logistic regression framework. The second part then modeled the variation in hospitalization outcomes, conditional on observing at least one hospital encounter. For this second part, we used generalized gamma distribution with log link function for cost and Poisson distribution with log link function for days and admissions.^5,6^ Normally-distributed random effects for the intercepts were incorporated in both parts and these effects were allowed to be correlated.^3^ Confidence intervals (95% CI) were estimated for all coefficients that depicted the ratio of dependent variable (e.g., per-period total cost) across independent variable categories (e.g., high-risk vs. standard-risk).

Independent variables in multi-variate regression models included sex, primary payer type at closest encounter to diagnosis, ALL risk (high vs. standard-risk), radiation vs. no radiation, BMT vs. no BMT, relapse vs. no relapse, binary variable indicating the first period and a linear term for all periods. Radiation was coded as “1” for the patient-period that was closest to the date of receiving radiation, and “0” otherwise. Since these patients are at a high-risk for continued hospitalizations, radiation was set as “1” for all subsequent periods. BMT and relapse were coded in the same fashion. Adjustments were also made for calendar year of diagnosis, end-of-life and timing of death in a period for patients who died. We also estimated these models without cancer outcomes (i.e., relapse, BMT and near death) and our overall results were qualitatively similar (not published).

**Table S1: Acute Lymphoblastic Leukemia Treatment Protocols followed by the Primary Children’s Hospital from 1998 to 2012.^a^**

| **Years** | **Children's Cancer Group (CCG)**  **Clinical Trials** | | | **Children's Oncology Group (COG)**  **Clinical Trials** | | | | | | | | |
| --- | --- | --- | --- | --- | --- | --- | --- | --- | --- | --- | --- | --- |
| 1998 | **1952**  SD: May’96 | **1961**  SD: Sep’96 |  |  |  |  |  |  |  |  |  |  |
| 1999 |  |  |  |  |  |  |  |  |  |  |  |  |
| 2000 | CD: Feb’00 |  | **1991**  SD: Jun’00 |  |  |  |  |  |  |  |  |  |
| 2001 |  |  | Single vs. double delayed intensification | **AALL00P2**  SD: Apr’01 |  |  |  |  |  |  |  |  |
| 2002 |  | CD: May’02 |  |  | **AALL0031**  SD: Oct’02 |  |  |  |  |  |  |  |
| 2003 |  |  |  |  |  | **AALL0232**  SD: Dec’03 |  |  |  |  |  |  |
| 2004 |  |  |  | CD: Oct’04 |  |  |  |  |  |  |  |  |
| 2005 |  |  | CD: Jan’05 |  |  | Double delayed intensification with high dose Methotrexate (MTX) | **AALL0331**  SD: Apr’05 |  |  |  |  |  |
| 2006 |  |  |  |  | CD: Oct’06 |  | All patients received PEG-asparginase |  |  |  |  |  |
| 2007 |  |  |  |  |  |  |  | **AALL0434**  SD: Jan’07 |  |  |  |  |
| 2008 |  |  |  |  |  |  |  |  | **AALL07P4**  SD: Jul’08 | **AALL0622**  SD: Jul’08 |  |  |
| 2009 |  |  |  |  |  |  |  |  |  |  |  |  |
| 2010 |  |  |  |  |  |  | CD: May’10 |  |  |  | **AALL0932**  SD: Aug’10 |  |
| 2011 |  |  |  |  |  | CD: Jan’11 |  |  |  |  |  |  |
| 2012 |  |  |  |  |  |  |  | CD: Jul’14 | CD: Sep’12 | CD: Feb’12 | Open | **AALL1131**  SD: Feb’12  Open |

^a^ SD and CD refer to starting date and closing date, respectively.

**Figure S1: Per-Patient First Year Hospitalization Cost for Pediatric, Adolescent and Young Adult Acute Lymphoblastic Leukemia from 1998 to 2012**

**Figure A: Median Costs^a^**

**
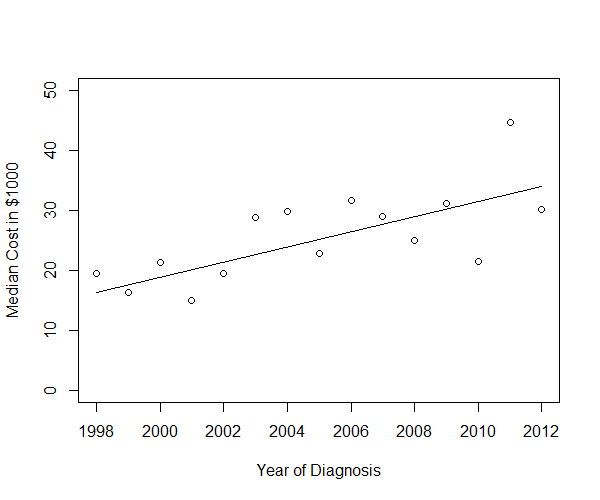
**

^a^ This figure describes the trend in the median hospitalization-related total cost incurred within the first year of cancer diagnosis by diagnosis years. We also show the linearly fitted regression line.

| **Figure B: Variable Costs^b^**  **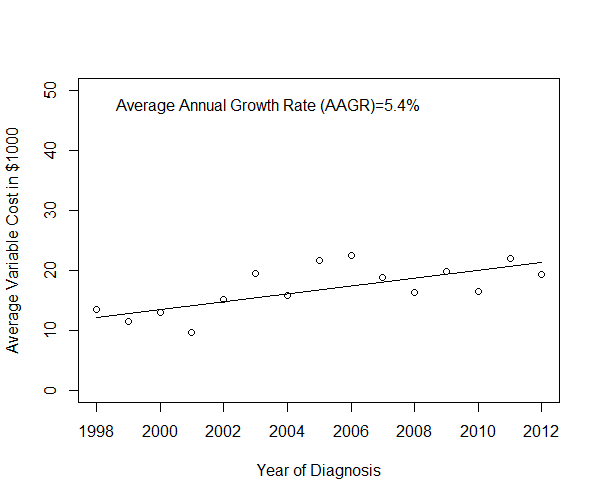** | **Figure C: Fixed Costs^b^**  **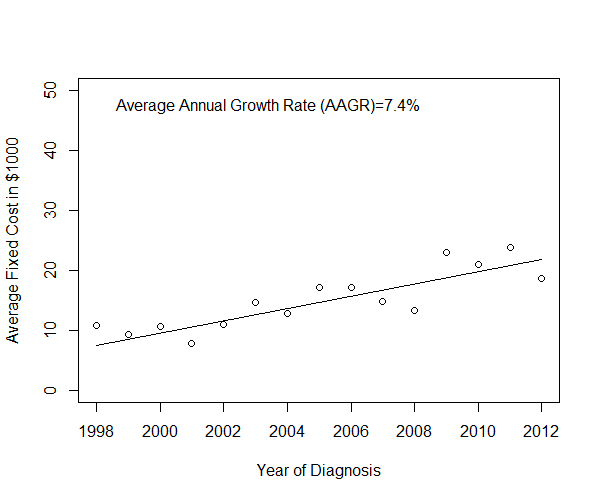** |
| --- | --- |

^b^ Average total hospitalization-related variable and fixed costs incurred within first year of cancer treatment by diagnosis year. Average costs were computed by dividing the first-year total costs of treating patients diagnosed in a year by the number of patients diagnosed in that year. We also show the linearly fitted regression lines.

**References**

1. Smith M, Arthur D, Camitta B, et al. Uniform approach to risk classification and treatment assignment for children with acute lymphoblastic leukemia. Journal of Clinical Oncology. 1996;14(1):18-24.
2. National Cancer Institute, General information about childhood acute lymphoblastic leukemia http://www.cancer.gov/cancertopics/pdq/treatment/childall/healthprofessional#Reference1. Accessed September, 2015.
3. Olsen MK, Schafer JL. A two-part random-effects model for semicontinuous longitudinal data. Journal of the American Statistical Association. 2001;96(454):730-745.
4. Spriensma A, Hajos T, Boer M, Heymans M, Twisk J. A new approach to analyse longitudinal epidemiological data with an excess of zeros. BMC Med Res Methodol. 2013;13(1):1-7.
5. Manning WG, Basu A, Mullahy J. Generalized modeling approaches to risk adjustment of skewed outcomes data. Journal of Health Economics. 2005;24(3):465-488.
6. Zou G. A Modified Poisson regression approach to prospective studies with binary data. American Journal of Epidemiology. 2004;159(7):702-706.
